# Perceptual Simulation in Gender Categorization: Associations between Gender, Vertical Height, and Spatial Size

**DOI:** 10.1371/journal.pone.0089768

**Published:** 2014-02-25

**Authors:** Xiaobin Zhang, Qiong Li, Kendall J. Eskine, Bin Zuo

**Affiliations:** 1 School of Psychology, Northwest Normal University, Lanzhou, China; 2 Department of Psychological Sciences, Loyola University, New Orleans, Louisiana, United States of America; 3 School of Psychology, Central China Normal University, Wuhan, China; University of Akron, United States of America

## Abstract

The current studies extend perceptual symbol systems theory to the processing of gender categorization by revealing that gender categorization recruits perceptual simulations of spatial height and size dimensions. In study 1, categorization of male faces were faster when the faces were in the “up” (i.e., higher on the vertical axis) rather than the “down” (i.e., lower on the vertical axis) position and vice versa for female face categorization. Study 2 found that responses to male names depicted in larger font were faster than male names depicted in smaller font, whereas opposite response patterns were given for female names. Study 3 confirmed that the effect in Study 2 was not due to metaphoric relationships between gender and social power. Together, these findings suggest that representation of gender (social categorization) also involves processes of perceptual simulation.

## Introduction

Emerging evidence has revealed that human thought draws from one's embodiment, which refers both to actual bodily states and to simulations of embodied experiences in the brain's modality-specific systems for perception, action, and introspection [1]. For example, Borghi, Glenberg, and Kaschak showed that verifying whether a given object (e.g., a car) had a certain part (e.g., a roof) involved perceptual simulation processes such that when participants' reaction movement (upward or downward) corresponded with the position of the part relative to the object (e.g., roof vs. upward), the reaction was quicker [2]. In another study, after watching an action cartoon, participants were asked to describe the cartoon to a listener when the cartoon was no longer present. The result showed that participants who were prevented from gesturing (keeping stationary) processed the cartoon's description significantly slower than control participants [3]. Similarly, Tucker and Ellis found representations of grapes and hammers can be activated through simulations of motor processes involved in precision and power grips, respectively [4].

Such findings can be explained through a perceptual symbol systems account, which undergirds such “embodied cognition” effects [1]. According to perceptual symbol systems theory, conceptual representations are tied to their perceptual basis and conceptual processing involves the partial simulation of those perceptual experiences that initially accompanied category exemplars. Perceptual symbols are raw materials that make up the variable constructions (i.e., simulations) and they draw from all senses, including proprioception, introspection, and motor programs [5], and they are derived from multiple sources of direct experience [6].

Many studies have shown that processing abstract and concrete concepts activates modality-specific simulation of physical space [5]. In one study, two words (e.g., root and branch) were presented above each other, and their order either followed the canonical arrangement (i.e., branch above root) or a contradictory arrangement (i.e., root above branch). Participants were asked to judge whether the two were related or not. The results showed that reactions were quicker when the arrangement of the words followed the canonical arrangement of the objects [7]. Some studies demonstrated that judging valence involves perceptual simulation of the vertical spatial dimension, on which good is up and bad is down [8], [9], [10], [11]. Other studies showed that the representation *TIME* involves the perceptual simulation of both horizontal space dimension [12], [13], [14], [15], [16] and spatial size dimensions [17], [18]. Along these lines, additional research has revealed that the representation and processing of *SOCIAL POWER* also recruits vertical spatial perceptual simulation such that power  =  up and powerless  =  down [5], [19], [20], [21]. Additional findings indicate that power was also represented in terms of size cues in which power  =  big and powerless  =  small [22].

It has been argued that gender is the most frequently utilized domain in human categorization [23], yet there is a sparse amount of research investigating its embodiment. Some research has shown that gender category (male and female) also be grounded in sensorimotor metaphors [24]. For example, Slepian and colleagues found that *MALE* was associated with the proprioceptive experience of “tough” and *FEMALE* was associated with the proprioceptive experience of “tender” [24], and the authors explained the results by the perspective that the largest trait difference between the male and female was the difference in tenderness [25]. In addition to traits like “tender” and “tough”, it can be argued that stature is another important and salient physical difference between males and females. On average, men are taller and larger than women. According to the perceptual symbols account, the representation of gender may involve the perceptual simulation of experiences rooted in physical dimensions of vertical height and spatial size. The purpose of the present research was to demonstrate that the processes involved with gender categorization activate perceptual simulations involved with spatial dimensions. Based on the above analysis, we hypothesized that the representation of gender (male and female) will also involve the processing of perceptional simulation such that *MALE* categories will be processed faster along lines of greater size and higher verticality and vice versa for *FEMALES*.

## Study 1

We conducted study 1 to determine if the representation and processing of gender involves the perceptual simulation of vertical dimension. We predicted that the male faces would be judged faster if they were presented at the top of the computer screen, but the female faces would be judged faster if they were presented at the bottom of the computer screen.

### Methods

#### Ethics Statement

This study was reviewed and approved by the committee for the protection of subjects at Central China Normal University, School of Psychology Ethics Committee. Written consent was also obtained from each participant before the experiment according to the established guidelines of the committee. This procedure was followed in studies 2 and 3 as well.

#### Participants and design

Participants were 43 Chinese undergraduates (23 females) each whom were offered a notebook as compensation. The study had a 2 (facial gender: male or female)×2 (position: top or bottom) repeated-measures design.

#### Stimulus, materials, and procedure

Eighty-eight white and black photographs of faces (44 males and 44 females) were selected on the basis of earlier prior testing. All picture files were standardized in size to 100×120 pixels, and all the faces displayed neutral expressions. Participants arrived to the laboratory individually and were greeted by a male experimenter. They were seated facing the computer screen and told that the study was investigating aspects of face perception. The procedure was the same as the one used by [8]. In each trial, a fixation cross was firstly presented at the center of the screen for 300 ms. Following this central cue, a subsequent fixation cross was flashed for 300 ms either at the 40% position (from top to down, above the central cue) or at the 60% position (from top to down, below the central cue) of the screen. A third fixation cross was flashed for 300 ms either at the 30% position (from top to down, above the central cue) or at the 70% position (from top to down, below the central cue) of the screen (in the same vertical direction as the second cross). The face pictures then appeared either at the 25% position (from top to down, above the central cue) or at the 75% (from top to down, below the central cue) position of the screen for 2000 ms (in the same vertical direction as the third cross), or disappeared when the participants made a response. The spatial cues (cross) were intended to direct attention to the spot of the picture's appearance, and thereby reduce random spatial exploration and additional error variance [8]. All of the fixations and pictures appeared centered horizontally on the screen. Participants were required to report, by means of a key press, whether each face depicted a male or female target as quickly and accurately as possible, and the response keys were counterbalanced across the sample. If the response was inaccurate, the word “incorrect” appeared in a red font for 1500 ms. Accurate trials were separated by a blank screen for 500 ms. On completion of the experiment, participants were debriefed and dismissed.

### Results and Discussion

**Figure 1:**
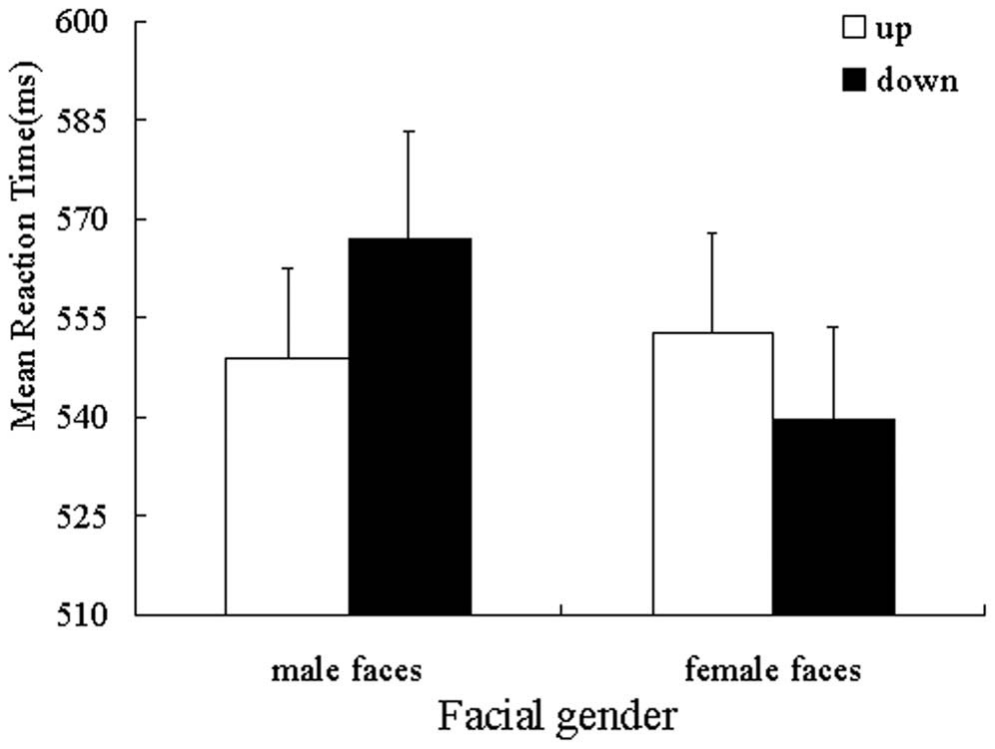
Mean reaction latency as a function of facial gender and position (study 1). Error bars Indicate standard error of the mean.

Mean categorization latencies served as the dependent measure of interest. Given the presence of extreme responses in the data set, response times that were slower than 2.5 standard deviations were excluded from the analysis, as were trials on which errors were committed [26]. This resulted in 0.09% (350) of the data being excluded from the statistical analysis. Latencies were then log-transformed to normalize their distribution [27]. For ease of interpretation, however, the untransformed means are reported in Figure 1. The transformed latencies were submitted to a 2 (facial gender: male vs. female) ×2 (position: top vs. bottom) ANOVA. The analysis revealed no main effects of position [*F*(1, 42) = 0.005, *p* = 0.94,
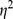
<0.001] and facial gender [*F*(1,42) = 2.690, *p* = 0.108,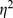 = 0.060]. As we expected, the facial gender × position interaction was significant [*F*(1, 42)  = 9.028, *p* = 0.004,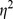 = 0.177; see fig. 1]. Simple effects analysis demonstrated that responses to male faces in the up position (*M* = 548.78, *SD* = 90.30) were significantly faster than in the down position (*M* = 566.85, *SD* = 108.48), [*F*(1, 42)  = 4.58, *p* = 0.038,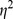 = 0.098]. Alternatively, responses to female faces in the up position (*M* = 552.58, *SD* = 99.71) were significantly slower than in the down position (*M* = 539.79, *SD* = 90.98), [*F*(1, 42)  = 5.30, *p* = 0.026, 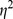 = 0.112].

The results of study 1 indicated that the representation of gender involved the perceptual simulation of vertical space. If the gender-linked spatial height (e.g., male face was presented on the top of screen) was consistent with the actual physical feature of gender, gender categorization was facilitated. If gender and spatial height were incongruent, gender categorization was degraded.

## Study 2

In addition to vertical height, size is also a salient physical difference between males and females. We conducted study 2 to determine if the representation and processing of gender involves the perceptual simulation of spatial size. We predicted that male names would be judged faster if they were presented in bigger font size, whereas female names would be judged faster if they were presented in smaller font size.

### Methods

#### Participants and design

Participants were 48 Chinese undergraduates (23 males) who were offered a notebook as compensation. The experiment had a 2 (name type: male names or female names) ×2 (name font size: big or small) repeated-measures design.

#### Stimulus, materials, and procedure

Eighty names (40 male names and 40 female names) were selected on the basis of earlier pilot testing. One hundred and twenty-two common names (66 male names, 66 female names, not including the names of famous people) were chosen from the internet, which 28 participants (14 males) rated on 7-point scales (extremely suited to female, “1” to extremely suited to male, “7”), “the extent the name can be used for male or female”. We then chose the highest scored 40 male names (the lowest score is 6.07, *M* = 6.44, *SD* = 0.219), and we chose the lowest scored 40 female names (the highest score is 1.36, *M* = 1.36, *SD* = 0.73). The scores of the male name were significantly higher than the female name, *t*(39) = 219.44,*p*<0.001. Finally, male names and female names were matched with the same 40 common family name.

Participants arrived at the laboratory individually and were greeted by a male experimenter. They were seated facing the computer screen and told that the study was investigated people's ability to classify names by gender. In each trial, a fixation cross was firstly presented at the center of the screen for 800 ms, at which point a name appeared at the center of the screen for 2000 ms or disappeared when the participants made a response. Twenty male (female) names were presented at the screen in large font size (70 point), and twenty different male (female) names were presented on the screen in small font size (25 point). The inter-trial interval was 250 ms. Participants were required to report, by means of a key press, whether each name was a male name or female name as quickly and accurately as possible, and the response keys were counterbalanced across the sample. If the response was inaccurate (slower than 2000 ms), the word “incorrect” (“please be quicker”) appeared in a red font for 1000 ms. On completion of the experiment, participants were debriefed and dismissed.

### Results and Discussion

**Figure 2:**
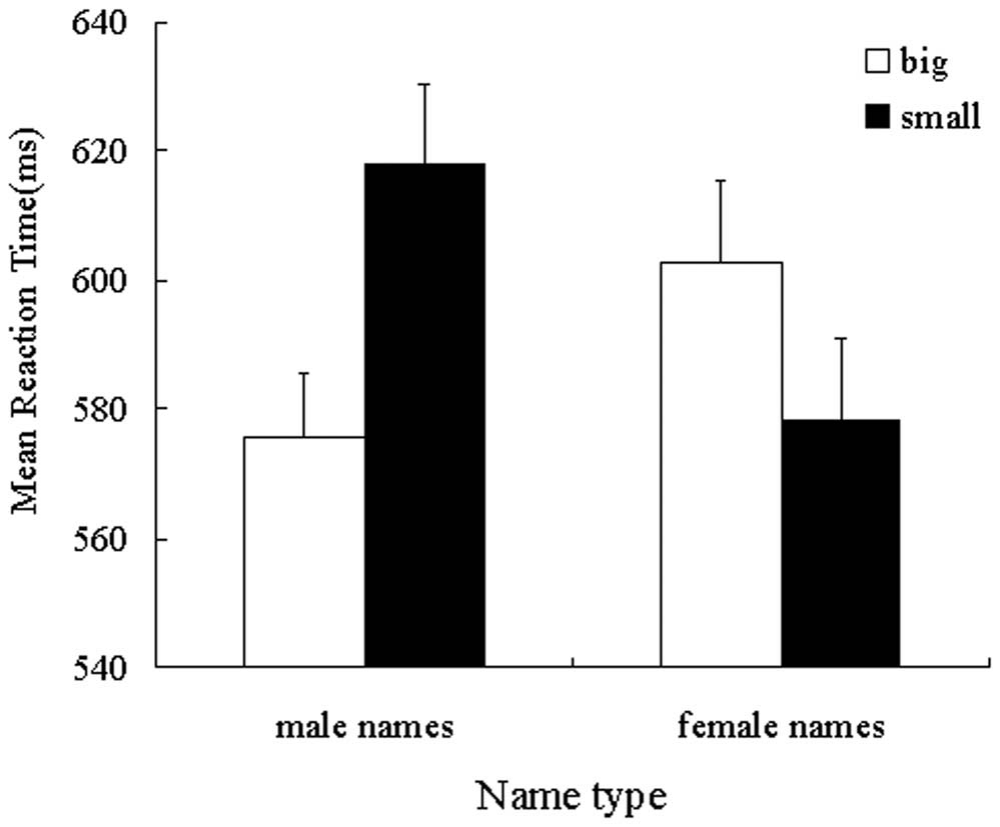
Mean reaction latency as a function of name type and name font size (study 2). Error bars indicate standard error of the mean.

Mean categorization latencies served as the dependent measure of interest. Given the presence of extreme responses in the data set, response times that were slower than 2.5 standard deviations were excluded from the analysis, as were trials on which errors were committed [26]. This resulted in 0.05% (191) of the data being excluded from the statistical analysis. Latencies were then log-transformed to normalize their distribution [27]. For ease of interpretation, however, the untransformed means are reported in Figure 2. The transformed latencies were submitted to a 2 (name type: male names or female names) ×2 (name font size: big or small) ANOVA. The analysis revealed no main effects of name gender [*F*(1, 47)  = 1.69, *p* = 0.20,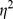 = 0.035]. The main effect of name size was significant [*F*(1, 47)  = 4.48, *p* = 0.04,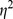 = 0.087], such that participants were faster to categorize big font size names (*M* = 589.20, *SD* = 78.90) than small font size names (*M* = 598.16, *SD* = 86.62). Most importantly, as predicted, the name type × name size interaction was significant [*F*(1, 47)  = 78.26, *p*<0.001,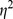 = 0.625; see fig. 2]. Simple effects analysis demonstrated that responses to big sized male names (*M* = 575.70, *SD* = 69.33) were significantly faster than small sized male names (*M* = 618.12, *SD* = 85.34), [*F*(1, 47)  = 57.30, *p*<0.001, 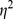 = 0.549]. Contrarily, responses to the big sized female names (*M* = 602.70, *SD* = 88.47) were significantly slower than small sized female names (*M* = 578.20, *SD* = 87.91), [*F*(1,47) = 22.44, *p*<0.001,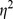 = 0.323].

The results of study 2 indicated that representation of gender also involved the perceptual simulation of spatial size such that male was associated with larger spatial size, and female was associated with smaller spatial size.

## Study 3

The results of studies1 and 2 suggest that representations of gender involved perceptual simulation processes of spatial height and size dimensions. In study 1, participants showed a congruency effect in categorizing male and female faces, high and low, respectively. In study 2, participants showed a congruency effect in categorizing male and female names, in large and small font, respectively. But these effects can also be driven by the metaphors of social power. According to the Chinese tradition culture, the power and social status of men were higher than women, and the men were thought to make greater contributions to society than women. Even now, men occupy more senior official careers than women in China. Until January 1, 2010, there were only three women in the 26 ministers of Chinese government departments, accounting for 11.5% (Institute of Women, the All China Women's Federation) [28]. Previous research has demonstrated that vertical positions were important metaphors of power, and the powerful was associated with up and the powerless was associated with down [5], [19], [20], [21], and mental representation of power was also associated with size cues [22]. Participants reacted faster when powerful groups appeared on top and powerless groups appeared at the bottom [5], and participants reacted faster when the names of powerful groups appeared in big size (compared to small size) and vice versa for powerless groups [22]. Based on the above analysis, the effect found in studies 1 and 2 may also result from the metaphors of power. To examine the possibility that these results are outcomes of metaphor endorsement, rather than perceptual symbols, in study 3 we recruited a group of participants who do not endorse the power-verticality metaphor for gender.

### Methods

#### Participants and design

Two hundred and two undergraduates (184 females, 18 males; age ranged from 18 to 24) were chosen randomly to complete Attitudes Towards Women Scale (AWS), a 25-item questionnaire [29]. The AWS measures traditional and conservative attitudes of women's place, including separate factors of rights, position relative to men, freedom, family role, and legal rights for college-aged participants [30]. The 25 items are measured on a scale ranging from 1 (strongly disagree) to 7 (strongly agree), where lower scores indicate more traditional, antifeminist views and higher scores indicate more positive and pro-feminist attitudes [31]. The AWS has been used in Korea, Taiwan, and China and was found to have good validity and reliability in these samples [32], [33], [34]. We then chose the 38 highest scoring undergraduates (37 females, 1 male, age ranged from 18 to 22) to complete the experiment. Their scores ranged from 141 to 173 (*M* = 148.86, *SD* = 5.99). The experiment had a 2 (name type: male names or female names) ×2 (name font size: big or small) repeated-measures design.

#### Stimulus, materials, and procedure

The procedure was the same as study 2.

### Results and Discussion

**Figure 3:**
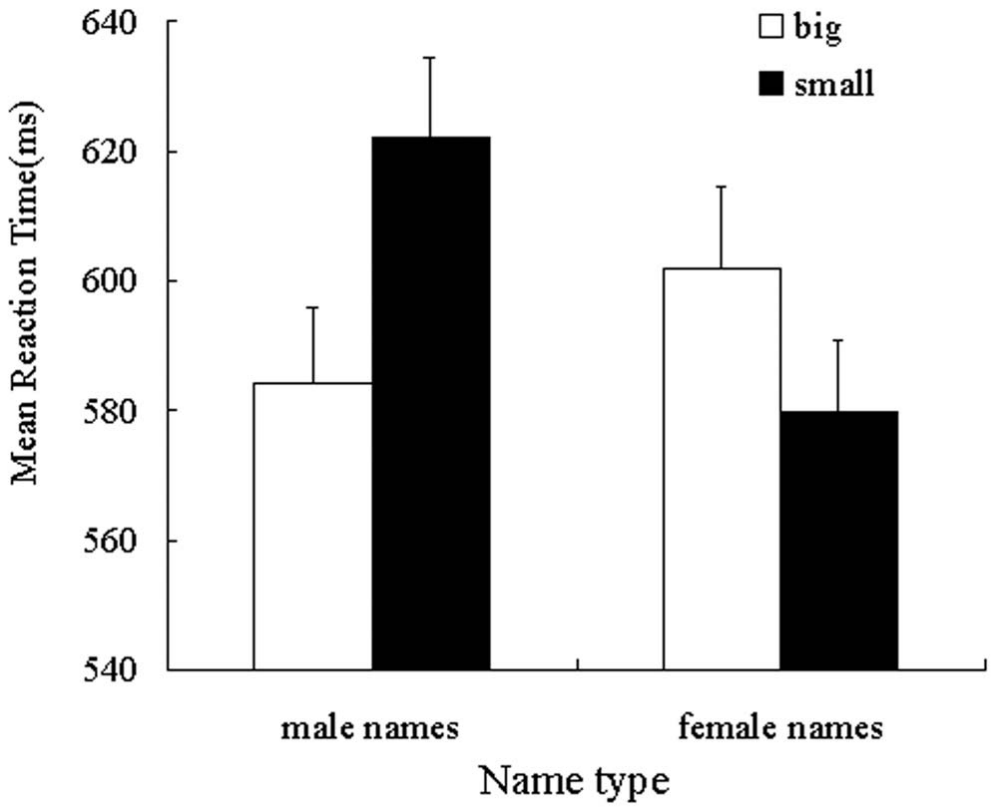
Mean reaction latency as a function of name type and name font size (study 3). Error bars indicate standard error of the mean.

Mean categorization latencies served as the dependent measure of interest. Given the presence of extreme responses in the data set, response times that were slower than 2.5 standard deviations were excluded from the analysis, as were trials on which errors were committed [26]. This resulted in 0.05% (139) of the data being excluded from the statistical analysis. Latencies were then log-transformed to normalize their distribution [27]. For ease of interpretation, however, the untransformed means are reported in Figure 3. The transformed latencies were submitted to a 2 (name type: male names or female names) ×2 (name font size: big or small) ANOVA. The analysis revealed no main effect of name font size [*F*(1, 37)  = 2.88, *p* = 0.098,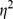 = 0.072]. The main effect of name type was significant [*F*(1, 37)  = 4.48, *p* = 0.04,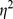 = 0.108], such that participants were faster to categorize female names (*M* = 590.69, *SD* = 74.15) than male names (*M* = 603.16, *SD* = 74.23). As in study 2, the name type × name size interaction was significant [*F*(1, 37)  = 42.19, *p*<0.001,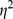 = 0.53; see fig. 3]. Simple effects analyses demonstrated that responses to big sized male names (*M* = 584.33, *SD* = 71.87) were significantly faster than smaller sized male names (*M* = 621.99, *SD* = 76.59), [*F*(1, 37) = 28.73, *p*<0.001, 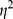 = 0.44]. Conversely, responses to big sized female names (*M* = 601.70, *SD* = 79.42) were significantly slower than small sized female names (*M* = 579.67, *SD* = 68.88), [*F*(1, 37)  = 12.20, *p* = 0.001, 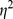 = 0.25]. Including AWS scores as a covariate, we repeated the above analysis as an ANCOVA. The analysis revealed no main effect of AWS scores [*F*(1, 36)  = 2.29, *p* = 0.67,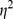 = 0.005]. The interactions between AWS scores and other factors were also not significant, *ps*>0.28.

The results of study 3 indicated that feminists, who do not endorse the power-verticality metaphor for gender, represent and process gender in similar spatial and size dimensions, thus confirmed that the effects reported in studies 1 and 2 are not a result of “social power” metaphors.

## General Discussion

A wide array of research now supports the view that human cognition is grounded in and shaped by sensorimotor experiences and that our conceptual representations include sensory, motor, and introspective activations that are recruited into partial simulations, which reenact various embodied states [35]. While previous research has revealed that gender-category representations include sensorimotor information related to handling hard and soft objects and proprioceptive experience (toughness) [24], no research to date has explored how salient gender differences in size affect the processing of gender representations. In the present research, we have provided evidence that the representation and processing of gender activates simulations involved with vertical height and spatial size dimensions, with *MALE* being associated with up and big and *FEMALE* being associated with down and small. Importantly, by recruiting feminist participants in a separate study and replicating the observed effects, we ruled out the alternative explanation that concepts involving “social power” might be driving these effects. These results are consistent with perceptual symbol systems theory by spotlighting how processing concepts can be affected by their perceptual basis [6], which we have extended to include the social categorization of gender in terms of vertical height and spatial size.

While the present findings could potentially be explained by either conceptual metaphor or perceptual symbol accounts, we proposed the latter approach appears to have the most explanatory power. First, it has been argued elsewhere that some metaphors (such as vertical position) are more basic than others [36]. One implication would be that basic metaphors (vertical position) are so prevalent because they draw from similar embodied experiences and thus use perceptual simulation in order to ground their meaning. That is perceptual simulation may be the basic of some basic metaphors [36]. Second, the results from the feminist participants suggest that metaphors are not being used, as they would naturally recruit different metaphors regarding size and gender. Third, metaphor serves to only make abstract relationships more concrete [37]. In actuality, males are on average taller than females. Additionally, on average, men are larger than women. Yet men being larger and taller than women are not abstract in this sense, and it can be seen directly and this correlation then, is based in concrete sensory experience. Finally, a perceptual account is more parsimonious and is better aligned with one's general empirical experiences in that men and women typically correspond to specific physical sizes.

Our data provide further evidence that early perceptual processes contribute to social-categorical thinking. Whereas prior research demonstrated that facial cues and visual acuity can affect the speed of social categorization [27], the studies presented here demonstrated vertical height and spatial size can also contribute to social-categorical thinking. Just as visual cues are present on every human face and are therefore likely to have a ubiquitous influence on categorical thinking about other people, spatial position and size are normally present for males and females and are therefore likely to have a ubiquitous influence on gender categorization. In particular, a person's position (e.g., sitting on a high or low stool) or stature (e.g., taking up more or less physical space) might influence how they are judged and potentially how they judge others in the social world.

Based on present studies, further research should further explore whether and how the present findings influence formation of specific gender stereotypes. The previous studies showed that the high position was associated with good and the low position was associated with bad [8]. We can infer that because processing the concept of male (female) involved perceptual stimulation associating with high (low) position which is relevant with positive (negative) valance respectively, the perceptual simulation involving gender categorization may play an important role in the formation of negative stereotypes towards women. For example, a lot of studies have shown that people had the stereotype that woman can not reach high achievements in mathematics [38], [39], [40]. Future research should explore the formation of this negative female stereotype by the perspective of representation of concept of gender (male and female) based on the present study. Further, it would be interesting to test whether inconsistent perceptual-gender properties (e.g., very tall women and very small men) influence social judgments in stereotype inconsistent ways. Specifically, additional research could explore if the activation of female stereotypes to (such as females are timid and so on) who are in high positions may be inhibited compared to females in low positions, and whether the activation of male stereotypes (such as males are rude and so on) who are in low positions may be inhibited compared to males in high positions.
